# Fabrication of aortic bioprosthesis by decellularization, fibrin glue coating and re-endothelization: a cell scaffold approach

**DOI:** 10.1007/s40204-019-00122-2

**Published:** 2019-10-12

**Authors:** Sonal Walawalkar, Shahdab Almelkar

**Affiliations:** HEALTH BIOLABS, Division of Tissue Engineering and Cell Science (TECS), Shree Hospital and Research Institute, Kolhapur, Maharashtra 416008 India

**Keywords:** Aortic aneurysm, Scaffold, Re-endothelization, vWF, SEM, Fibrin glue

## Abstract

Aortic dysfunctions (aneurysm, aortitis) lead to the most serious conditions related to aortic wall with life-threatening complications. The most common modality of management for such conditions is replacement (diseased part) of aorta by a larger diameter stent (reconstructive vascular surgery) which in itself is a big trial. The most natural way is to use a re-endothelized scaffold. Developing a scaffold with biomimetic properties is an experimental aim for most of the scientists and surgeons. We aim to structure a strategy to overcome the well-known problems associated with aorta. In this study, we plan to remold a larger diameter blood vessel such as aorta from xenogeneic origin using different protocols to decellularize and comparing them with normal aorta. The chemicals and enzymes used for bovine aorta decellularization are 1% SDS (group II), 70% ethanol + 0.25% trypsin (group III), 70% ethanol (group IV), and 0.25% trypsin (group V). Group I served as control (without decellularization). Histology and SEM study were conducted for cellular presence/absence in all scaffolds. Later, the scaffolds were coated with the fibrin glue (FG) and endothelial cells were proliferated over them. 3D images were taken showing the remolding of the endothelial cells on FG-coated surfaces. The re-endothelization was confirmed by lectin and vWF^+/+^ expression. Graft elasticity and burst pressure were confirmed by biomechanical tensile testing. Further, the absence of host tissue DNA and presence of cellular DNA after re-endothelialization were confirmed by PicoGreen assay. The acceptability for metabolically active cellular proliferation on scaffolds and its non-toxicity were proved by cell viability assay. Current findings accomplish that larger diameter aorta extracellular matrix scaffold (group II) can be fabricated and re-endothelialized to develop non-thrombotic surfaces with improved graft patency with promising results compared to other fabricated scaffold groups.

## Introduction

The aorta is the largest blood vessel in the body. It arises from the left ventricle of the heart and is divided into four parts: the ascending aorta, aortic arch, thoracic and abdominal part of descending aorta. It is almost a few centimeters (cm) long and has a circumferential diameter of approximately 2.54 cm. The wall of aorta, like any other artery has three layers. The intima, the innermost layer (basal lamina) provides a smooth surface for blood to flow across. The media, the middle layer with smooth muscles comprising elastic fibers that allows the aorta to expand and contract with each heart beat. The adventitia, the outermost layer is mainly rich in fibroblast as well as in collagen zone, it provides additional support and structure to the aorta (Tran et al. 2018). The aortic vessels bear the pressure exerted by blood for an average of 120 mm (110–130 mm) of Hg in systole and 80 mm (70–90 mm) of Hg in diastole. The difference (pulse pressure) is approximately 40–50 mm of Hg (Narayan et al. 2017). Multiple diseases and vascular complications can cause damage to an aorta and put patients at great, life-threatening risk. Those conditions include atherosclerosis, stenosis (hardening of the arteries), hypertension, Marfan syndrome (genetic disorders), Ehlers–Danlos disorder (connective tissue alterations), Turners syndrome and injury. The basic pathology is disruption or denudation of endothelial lining leading to cascade of events which ultimately form atheroma and atherosclerosis. The wall becomes weaker and weaker leading to complications like stenosis, aneurysm, arteritis, etc.

An aortic aneurysm is an enlargement (dilation) of the aorta to greater than 1.5 times normal size. They usually cause no symptoms except when ruptured. Aortic aneurysm is generally found at abdominal part of the aorta, though thoracic part involvement is not uncommon. It is most commonly associated with aged population (60–75 years). The aorta becomes weak, dilated, bulged or sometimes may even burst. Annually approximately 150 thousand deaths occur due to rupture of aortic aneurysm all over the world. Another devastating aortic disease is an aortitis which encompasses inflammatory reactions in the aortic vessel wall associated with histopathological lesions (Starzl 1998). While inflammation can occur in response to any injury, including trauma, the most commonly known causes are infections or connective tissue disorders. Initial infection leads to non-infectious vasculitis by activating immune complexes and by cross-reactivity (Takayasu arteritis) (Zorger and Stelnbauer 2016). There is also reactive oxygen species (ROS) alteration. Inflammation of the aorta can cause aortic dilation, resulting in aortic insufficiency (Starzl 1998).

Once the complication establishes and advances to such an extent that it becomes impossible to manage them only on medical line, it becomes essential to treat it surgically. Current cardiovascular surgeries involve the replacement of the diseased aorta with a suitable graft as conduit. The success of the surgery depends upon both the properties of the graft and the surgical skills (Starzl 1998). For obvious reasons, the conduit must possess all the qualities of normal blood vessel (biomimetic properties) and should be accepted by the host. It must also be long lasting and should not give rise to any complications. Researchers have invented synthetic prosthesis. Biomaterials with high flow large porosity have been used successfully, but with low flow small porosity are not much promising (Harrison 1958). Till date widely used prosthetic grafts are expanded-poly(tetrafluoroethylene) (ePTFE) (Campbell et al. 1975). They act as a non-leaking polymer-based conduit, but there are many lacunae. These problems with the synthetic prosthesis are as follows: faulty sealing of the anastomosed edges of the graft to the normal aortic wall causing instability of the anatomy (the transition from the normal wall to the graft zone is not stable and smooth), layering of endothelial lining over the graft is impaired, endograft migration, graft kinking, endo-leak (types I, II, III, IV), graft thrombosis, endo-tension, and being expensive (Greenwald and Berry 2000).

To combat these defects, the researchers made use of xenogeneic tissue (bioprosthesis) and human tissue (homograft). The findings in the area of biomaterial fabrication have proved that extracellular matrix (ECM) scaffolds have improved compliance in comparison to synthetic prosthesis (Taylor et al. 1990). The synthetic biocompatible blood vessel grafts hardly become adsorbed as a blood vessel tissue matrix. Hence, the roles of biological source blood vessel are appreciated (Hansen 2005). The bioprostheses are usually structured in three steps: (a) scaffold formation, (b) delivery of tissue-inducing substrates and (c) seeding of the cells. The scaffold fabrication is the structuring of the matrix which will make the graft skeleton. It should have the following properties such as high porosity and proper pore size, high surface area, biodegradability (the degradation rate must match the new tissue formation). The fabricated scaffolds should also have the mechanical integrity to maintain the predesigned tissue structure and must positively interact with the cells including enhanced cell adhesion, growth, migration and differentiation. Fabrication of scaffolds must be biocompatible and non-toxic and support cellular migration, infiltration and plasticity. These properties are met better toward bioprosthesis and homografts compared to synthetic ones. There are various ways to structure the bioprosthesis. Decellularization is the vital step in making biological scaffold. The protocols of decellularization are numerous by employing the use of various chemicals and enzymes. Thus, with this aim, we planned to fabricate a larger diameter decellularized bovine aorta by using 3 ingredients. We used 1% SDS, 0.25% trypsin and 70% ethanol in different combinations to observe which method of decellularization works best.

To form a non-thrombotic surface on the bioprosthesis and to avoid the graft versus host reaction, the most natural way is to expand the endothelial cells on the luminal surface, i.e., endothelization. The researchers have attempted to layer the endothelium but failed to retain them in the luminal wall. The main challenge is to retain the endothelial layer under pulsatile sheer stress of blood flow on the basal lamina of the synthetic graft. Keeping this in consideration, we further re-endothelized the basal lamina of the decellularized bovine aorta (BA) on the fibrin glue matrix, so as to fabricate a non-thrombotic xenogeneic vessel. Later, the growth of endothelium over the scaffold and tightness of the endothelial junctions were checked so as to see the chances of wearing off of the endothelial layer. The whole experiments were documented and images were taken. The imaging of cells on the scaffold is a very difficult task. Using the image J software for construction of 3D images was useful in understanding the texture, orientation of the cells in tissue matrix and their interaction (Schneider et al. 2012). A 3D image construction for histological and SEM imaging provided a concrete vision for cellular behavior with respect to extracellular matrix and the information about the cell remolding on the scaffold. In this study we also showed the proliferation of cells and increase in their DNA on the cells expanded on the scaffolds. This study promises a future hope towards fabricating the various biological origin xenogenic or allogenic large diameter vascular bioprosthesis.

## Experimental

### Materials and methods

#### Ethical approval

All protocols were approved by Heal^th^ Biolabs, Shree Hospital Research Ethics Committee (SHREC), Kolhapur, India.

Sample collection: Bovine aorta and sheep external jugular vein were collected in a careful cold cell culture medium (4–8 °C) (Hi-Media, Mumbai, India) from local abattoir and transported to Division of Tissue Engineering and Cell Science (TECS) cell culture laboratory for processing.

#### Bovine aorta decellularization (scaffold grouping)

We have grouped bovine aorta (BA) (*n* = 5) as non-decellularized (control) group I. The samples were decellularized using different chemicals and cell dislodging enzymes. The decellularized groups were divided as follows: group II (1% SDS) *n* = 5, group III (70% ethanol + 0.25% trypsin) *n* = 5, group IV (70% ethanol) *n* = 5 and group V (0.2% trypsin) *n* = 5. All BA samples were incubated at 40 °C for 48 h in an incubator shaker for decellularization. All groups of BA samples were washed until neutral pH was achieved. The samples were fixed in 10% formaldehyde solution and were paraffin embedded. The paraffin blocks were trimmed, sectioned (5 µm) on fully automatic rotary microtome (Leica) (Steffed and Luscher 2007).

### HE-staining and histology of scaffolds

All the groups of BA samples such as group I, group II, group III, group IV and group V were examined for tissue histology. Hematoxylin (1%) staining was done for 20 min and immediately followed by 1% acid alcohol treatment. The sections were counter stained with eosin (1%). Sections were mounted in DPX and observed under phase contrast microscope for the cellular organization in the various tunicae of BA (intima, media and adventitia) (Carl Zeiss Meditec AG, Jena, Germany) (Steffed and Luscher 2007).

### Determination of cellularity

All nuclei in the intima, media and adventitia present within a band width were counted (Zeiss imaging system canon 32 pixel). Quadrants in three transverse sections (total of 12 counts) were averaged for each fresh control (*n* = 5) and decellularized (*n* = 5 each) specimen. Percentage decellularization was calculated using formula (Schaner et al. 2004; Almelkar et al. 2013a, b, c):

$${\text{Decellularization }}(\% )\, = \,\frac{{[{\text{cell count for fresh HSV}} - {\text{cell count decellularized HSV}}]}}{{{\text{cell count for fresh HSV}}}}\, \times \,{\text{1}}00.$$

### Extracellular matrix (EVG staining)

Collagen and elastin were analyzed similarly by fixing all groups BA (*n* = 3 each) samples in 10% formalin. Paraffin embedding was performed and transverse sections (5 µm) were taken. All the sections were stained by Verhoeff–van Gieson (EVG) method. The collagen and elastin-covered area in the stained sections were observed (Almelkar et al. 2013a, b, c).

### Fibrin glue formulation

It was performed in three steps as follows (Almelkar et al. 2014).

#### Step I: preparation of fibrinogen

10 mL of bovine blood was collected by external jugular venous puncture in a heparinized centrifuge tube. The blood sample was centrifuged at 2500 rpm for 10 min and separated plasma was stored at – 20 °C till further use.

#### Step II: preparation of thrombin

1 mL of fresh frozen plasma (FFP) was thawed at 2–4 °C. FFP was further diluted with distilled water (1:10) making the volume of 10 mL. 0.1 mL of 1% acetic acid was added to make pH 5.3. Addition of acetic acid led to formation of precipitate after 30 min incubation. Further, centrifugation at 2000 rpm was done for 5 min and the precipitate was collected. Normal saline (10 mL) was added and pH was achieved to neutral by addition of sodium carbonate. Further, incubation was done at 37 °C for 5 min and 0.1 M calcium chloride (CaCl_2_) was added. The clot formed in 45–120 s was removed leaving behind a transparent thrombin solution.

#### Step III: fibrin glue (FG) formulation

Fibrinogen 50 μL and thrombin 50 μL were mixed well on a tissue culture plate surface and plated on group II (1% SDS) BA scaffold. Further, the fibrin glue (FG) mixture was allowed to react and form meshwork sealant layer.

### Cell seeding

One end of the sheep external jugular vein (SEJV) was clamped. 0.2% collagenase type IV (Sigma Aldrich, St. Louis, MO, USA) and 0.25% dispase II (Roche, Nutley, NJ, USA) was injected in luminal cavity of the SEJV for dislodging of endothelial cells (ECs). Entire vein was incubated for 20 min at 37 °C. PBS medium was flushed through the SEJV luminal cavity and the digest was centrifuged at 1000 rpm for 10 min. Cell pellet was re-suspended in 10% endothelial conditioning growth medium-2 (ECGM-2) (Promo-Cell GmbH, Germany). SEJVECs were plated in tissue culture flasks (FB-coated and non-coated surfaces). At the end of 48 h ECs were fed with complete ECGM-2 containing 10% FBS, 2 mM l-glutamine. The SEJVECs were maintained till five passages. SEJVECs were also expanded on FB-coated group II (1% SDS) BA scaffold (Baudin et al. 2007).

### Immunocytochemistry for von Willebrand factor (vWF) expression

For immunocytochemistry, SEJVECs were grown on group II (1% SDS) scaffold and were fixed using 4% paraformaldehyde solutions. The cells were permeabilized (50% methanol) and blocked by 5% bovine serum albumin (BSA). The cells were then exposed to anti-von Willebrand factor (vWF) primary non-labeled antibody IgG fraction of antiserum developed in rabbit (1:200 dilution) (Sigma Aldrich, USA) for 12 h at 4 °C, followed by respective secondary antibodies (Sigma Aldrich, USA) for 1 h at 37 °C. The coverslips were mounted in anti-fade (Vecta-shield, Vector Laboratory, and Burlingame, CA) medium. The slides were examined on confocal laser scanning microscope—LSM 510 Zeiss workstation (Carl Zeiss Meditec AG, Jena, Germany). Negative control did not show any staining (Almelkar et al. 2013a, b, c; Wagner et al. 1982).

### Immunocytochemistry for lectin expression

Expanded SEJVECs on group II (1% SDS) BA scaffold were fixed in 4% paraformaldehyde (Himedia, Mumbai, India) and stained by FITC-conjugate *Lycopersicon esculentum*^+/+^ stain (lectin positive) prepared 2 μg/mL (1 h) (Zeiss, Apotome fluorescent microscope) (Favre et al. 2003).

### Assessment of cell attachment and morphology

All groups scaffold samples were fixed in 2.5% glutaraldehyde (2 h) and dehydrated with graded ethanol for 10 min. The samples were dried overnight and sputter coated with platinum (Quorum Technologies Ltd, UK). SEM (Zeiss Scanning Electron Microscope) was done for the decellularized BA scaffolds: group II (1% SDS) group III (70%-ethanol − 0.25% trypsin), group IV (70%-ethanol) and group V (0.2% trypsin). SEJVECs were expanded onto the lumenal surface of only group II (1% SDS) BA scaffold (Gulbins et al. 2006).

### Image J software for 3D construction

Image J software was used for construction of histological. A 3D construction and imaging for histology (HE and EVG staining) was obtained for groups I, II. This construction was also done for the FG-coated surfaces, endothelial cell expansion on FG-coated and non-coated surfaces. FG-coated lumenal surface on group II (1% SDS) scaffold as well as the non-coated lumenal group II scaffold was also done.

SEJVECs were expanded on FG-coated surface on group II (1% SDS) scaffold. A comparative approach was tried to develop between the non-3D and 3D constructed images (Schneider et al. 2012).

### Biomechanical tensile testing

A strip of BA (*n* = 5) was cut so that lumen formed the test specimen BA specimens from groups I and II. Group IIA and B which served as recellularized scaffold were kept wet with PBS. Stress/strain testing was performed with a Shimadzu AGS 500 G stress/strain tester (load cell of 500 g) with constant tissue strip length of 10 mm between the two clamps. The cut strip was enclosed in sand paper strip with adhesive glue, and the Young’s modulus gf/mm^2^, and burst pressure were examined by intraluminal pressure which was increased by 300 mm Hg/s using an ACS Indeflator plus 20 pressure gauge until vein rupture. The burst strength was recorded in mm Hg for both fresh control (*n* = 5) and decellularized (*n* = 5) specimens (Patwardhan and Vaideeswar 2004).

### PicoGreen DNA quantification

The samples in groups I, II, IIA and IIB (*n* = 5) were placed in lysis buffer for 15 min and macerated. 30 μL of each sample was obtained in 4 mL cuvette and 2.96 mL of distilled water was added followed by DNA samples incubation for 10 min. The absorbance was measured at 260 nm with ds-DNA levels quantified by a PicoGreen fluorescent assay to confirm the occurence of residual tissue ds-DNA in decellularized group. All samples were compared to group I. However, for cellular migration on BA scaffolds (biocompatibility) and re-endothelization of BA, PicoGreen assay was done to measure the presence of cellular DNA (Frank 2010).

### MTT for SEJVECs proliferation in culture and BA scaffold

MTT [3-(4,5-dimethylthiazol-2-yl)-2,5-diphenyltetrazolium bromide] (Himedia, Mumbai, India) (5 μg/mL) cell-based biochemical assay was performed for confirmation of SEJVECs proliferation on the surface of group IIA scaffolds (15,000 cells/BA scaffold) and tissue culture plate (20,000 cells/well). MTT assay was performed after 72 h for SEJVECs expansion by approximately 1 cm in length from group IIA and B (1% SDS-48 h) BA scaffold (*n* = 5) (Almelkar et al. 2013a, b, c).

### Statistical work

GraphPad Prism 5 software was used to obtain mean ± SD and one-way ANOVA (***p < 0.05) test was performed and graph was also obtained.

## Results

### Histology for cellular and matrix evaluation

Group I (control) BA was examined for histological details in various regions of aorta tunicae. The nuclei were stained dark blue black (HE staining) in all the tunicae in presence of a defined pinkish matrix. The ECM evaluation [Verhoeff–van Gieson (EVG)] showed the presence of dark black and reddish appearance texture. Black color elastic laminae and elastic fibers along with bundles of collagen dark red in color were seen. Group II (1% SDS) showed the absence of cells and nuclei from all the tunicae (HE staining) along with preservation of ECM. Further details on all groups and their histological findings are presented in Table 1.

**Table 1:** Histology for cellular and matrix evaluation in all four groups of BAS (bovine aorta scaffold) as compared to control

BA groups (cells)	Hematoxylin–eosin (HE)			BA groups (ECM)	Verhoeff–van Gieson (ECM)		
	Tunica adventitia	Tunica media	Tunica intima		Tunica adventitia	Tunica media	Tunica intima
I (Figure 1)	Fibroblast^(+/+/+)^ (dark blue-black nuclei).	SMCs^(+/+/+)^ (dark blue-black nuclei)	Endothelium^(+/+/+)^ (dark blue-black nuclei)	I (Figure 6)	Collagen^(+/+/+)^	Elastin^(+/+/+)^	Basal lamina with numerous invaginations or caveolae
II (Figure 2)	Fibroblast^(−/−)^	SMCs^(−/−)^	Endothelium^(−/−)^	II (Figure 7)	Collagen^(+/+/+)^	Elastin^(+/+/+)^	Basal lamina with numerous caveolae
III (Figure 3)	Fibroblast^(−/−)^	SMCs^(−/−)^	Endothelium^(−/−)^	III (Figure 8)	Collagen^(+/+)^	Elastin^(+/+)^	Intact basal lamina
IV (Figure 4)	Fibroblast^(+/−)^	SMCs^(−/−)^	Endothelium^(−/−)^	IV (Figure 9)	Collagen^(+/+)^	Elastin^(+/−)^	Intact basal lamina
V (Figure 5)	Fibroblast^(+/−)^	SMCs^(+/−)^	Endothelium^(+/−)^	V (Figure 10)	Collagen^(+/+)^	Elastin^(+/−)^	Intact basal lamina

*Absent (−/−), present/absent (+/−), moderately present (+/+), extensively present (+/+/+)

### Cellular determination

The various percentages of decellularization were observed in each and every group even though their time interval was kept constant (Table 2; Fig. 7). Maximum percentage of cellular decellularization was seen for group II (1% SDS). Hence on the basis of maximum percentage, decellularization group II (1% SDS) was selected for further cellular expansion studies (Table 2; Fig. 7).

**Table 2:** Percentage of decellularization

Methods	% Decellularization	*p* value	Cells (%)
Group I	0		100
Group II	98.33 ± 0.5	***p < 0.05	1.67
Group III	92.33 ± 2		7.67
Group IV	71 ± 7.25		29
Group V	58.67 ± 7		41.33

### Fibrin glue reaction

A transparent thrombin solution when it was reacted with fibrinogen gave meshwork of fibrin matrix (glue) on culture plate (Fig. 3b) as well as on group II (1% SDS) BA scaffold (Fig. 6e) in 20 s.

### SEJVECs on FG-coated surfaces and characterization

SEJVECs were cultured on non-coated and fibrin glue (FG)-coated surfaces. On non-coated surfaces the SEJVECs reached 70% confluency (157,000 cells/well) on day 10 while the cells expanded on FG-coated surface reached confluence on 5th day (391,500 cells/well). The SEJVECs resembled cobble stone appearance on non-coated surface (Fig. 3a). The SEJVECs, expanded on FG-coated surfaces, showed flatting and expansion of filopodia along with firm adherence (Fig. 3c). The SEJVECs were also expanded and on group II (1% SDS) BA scaffold, coated with FG, showed a von Willebrand factor (vWF^+/+^) (Fig. 5d) and *Lycopersicon esculentum*^+/+^ lectin (lectin positive) (Fig. 5b) expression.

### SEM studies for scaffolds

Group II (1% SDS) showed absence of cells from basal lamina (lumenal surface) with a preserved matrix without tissue injury (Fig. 6a). Group III (70% ethanol–0.25% trypsin) also showed intact basal lamina (lumenal surface) without alteration of matrix (Fig. 6b). Group IV (70% ethanol) also showed absence of cells from basal lamina with invagination. Alteration of the matrix was not observed (Fig. 6c). Group V (0.2% trypsin) showed damaged basal lamina (lumenal surface) and absence of cells. The matrix texture was also altered (Fig. 6d). Group II (1% SDS) BA scaffold clearly showed the fibrin glue meshwork with inter-nodal spaces (Fig. 6e). The re-endothelization (SEJVECs) was seen on FB-coated group II (1% SDS) scaffold (Fig. 6f).

### Image J (3D construction of pictures) a comparative approach (Table 3)

Image J 3D construction was done for the HE and EVG-stained groups. Histological images (HE and EVG) construction in 3D revealed the organization of cells in the blood vessel tissue matrix (tunicae) for group I control (Fig. 2a, f). The decellularized groups II (1% SDS) scaffold showed the absent of cells in all the matrix tunicae (Fig. 2b, g). The phase contrast imaging and 3D image J construction for SEJVECs, proliferated on FG-coated surfaces, showed flattening of the filopodia as well as the tightly joining of cell cluster (Fig. 4c). A non-coated surface showed proliferation without spreading (Fig. 4a). The 3D construction of phase contrast FG-coated surfaces showed inter-nodal space and arrangement of the meshwork fibrils (Fig. 4b).

**Table 3:** SEJEVCs in culture (non-coated and FG-coated), group II scaffolds and Image J analysis

Experiment	Culture	Group II
SEJVEC + non-coated	Cobble stone phenotype Proliferation without spreading (Figs. 3a and 4a)	Nil
FG coated	FG meshwork (Figs. 3b and 4b)	FG meshwork and inter-nodal space (Fig. 6e)
SEJVEC + FG coated	Flattening of cells along with expansion of filopodia (Figs. 3c and 4c)	Expansion of EC and remolding of cells as per matrix and luminal surface (Fig. 6f)

**Fig. 1:**
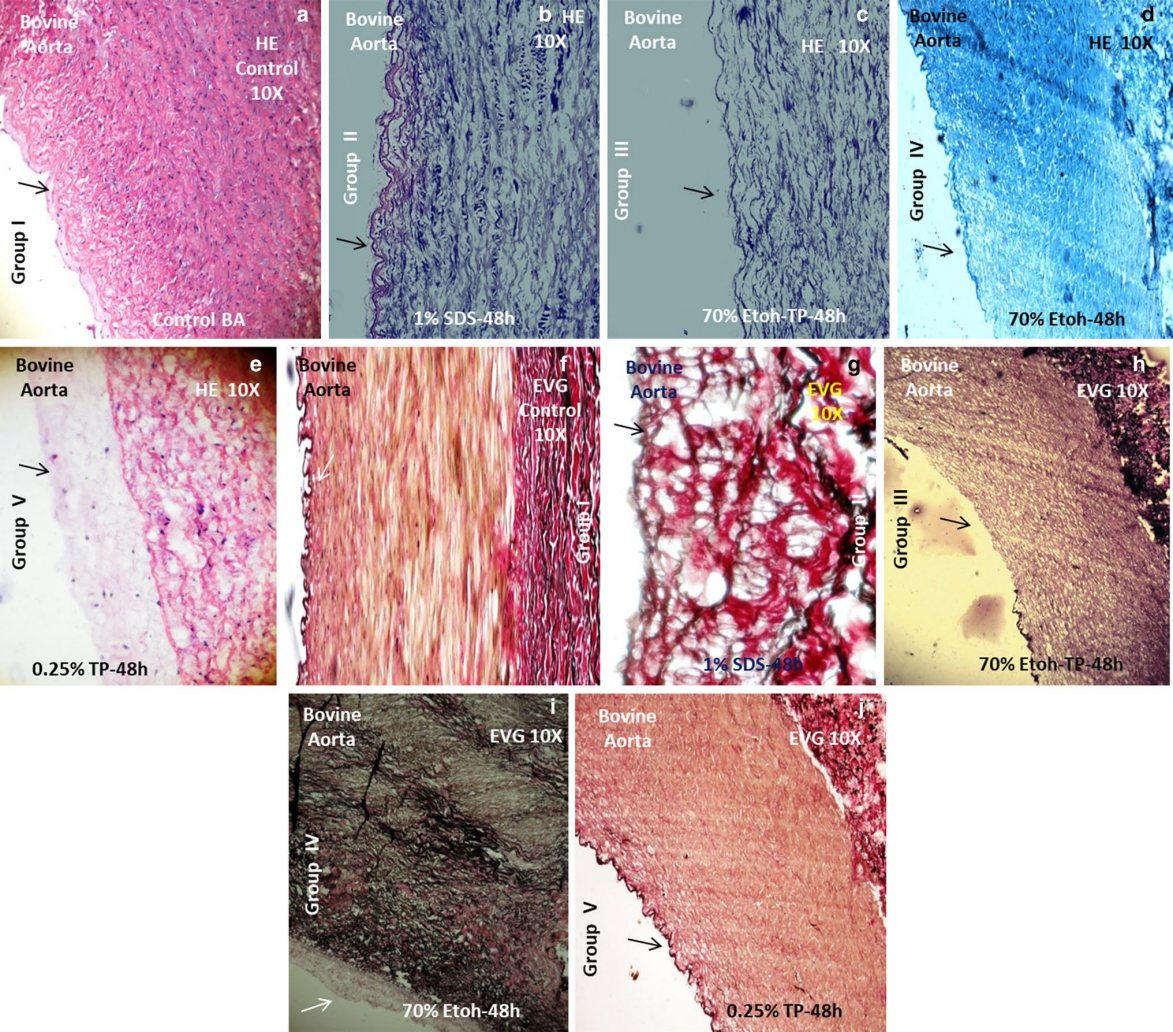
Histology. Group I (**a**) all tunicae–intima (EC^+/+/+^), media (SMCs^+/+/+^) and adventitia (FB^+/+/+^) shows presence of cells. While tunicae with invagination of basal lamina shows complete absence of cells for group II and III^**(**−/−/−)^ (**b**, **c**). In group IV cells were absent from intima^(−/−)^ and media^(−/−)^ junctional area. Few cell occurence in the junctional region of media^(+/−)^ and adventitia^(+/−)^ (**d**). Group V shows presence of scattered cells^(−/+)^ in all the tunicae (**e**). However, elastin and collagen staining for group I shows presence of elastic fibers^(+/+/+)^ and collagen^(+++)^ bundles (**f**). ECM evaluation for group II showed elastin (black)^**(**+/+/+)^ and collagen (red)^**(**+/+/+)^ (**g**). Group III shows elastin^(+/+)^ in media and collagen^(+/+)^ in adventitia and invagination in basal lamina (intima) (**h**). Group IV shows presence of fragmented elastic^(+/−)^ fibers along with collagen^(+/+)^ in adventitial matrix (**i**). The fragmentation of elastic tissue in media was observed in group V (**j**)

**Fig. 2:**
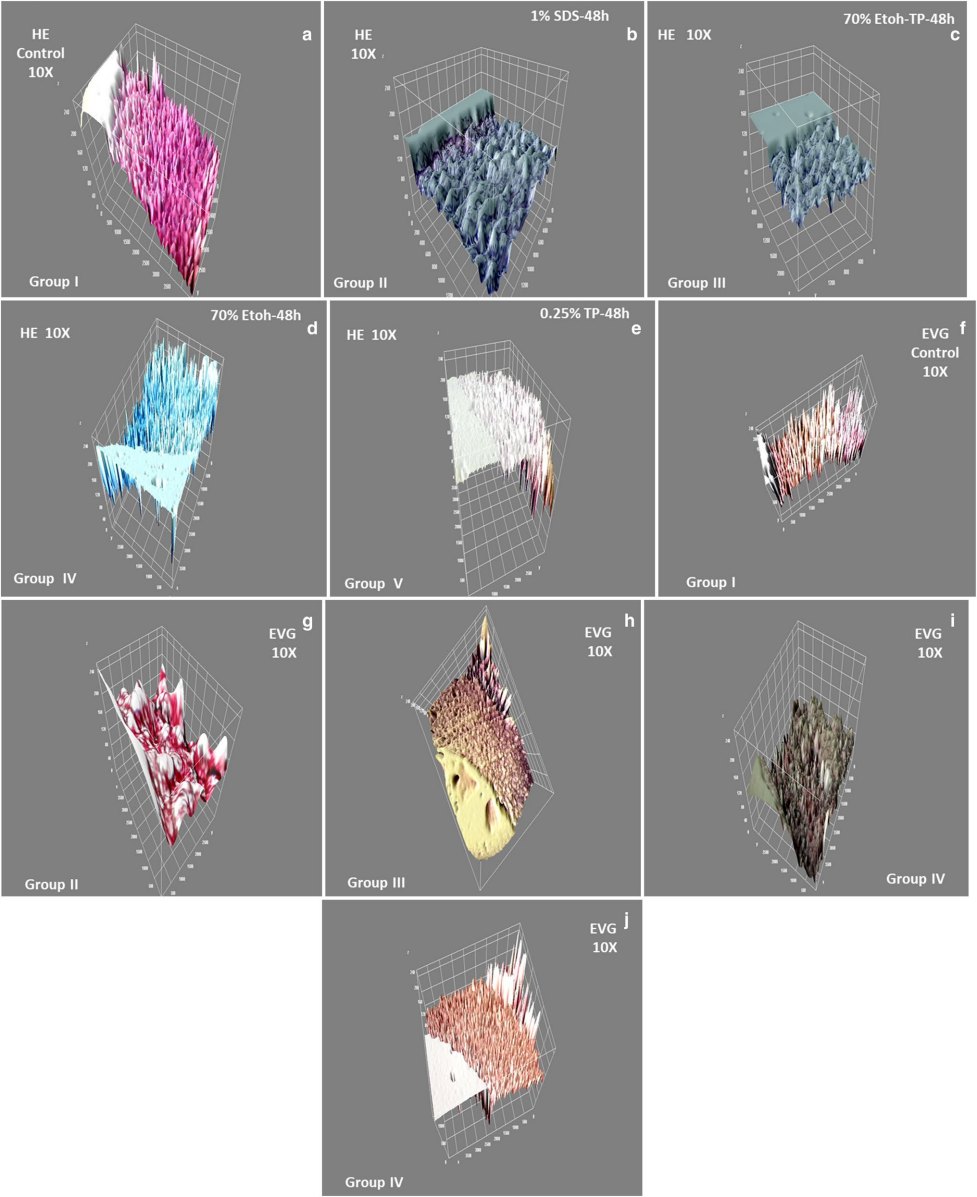
Image J 3D construction. Image J 3D construction for HE (**a**) and EVG staining (**f**) for group I showed cellular alignments on the elastic^(+/+/+)^ and collagen^(+/+/+)^ fibers with intact preservation of matrix. Group II scaffold in 3D shows absence of cells^(−/−/−)^ (**b**) with only presence of ECM such as elastic^(+/+/+)^ and collagen^(+/+/+)^ (**g**) bundles. Group III scaffold showed devoid of cell^(−/−/−)^ (**c**) with left over elastic fibers^(+/+)^ and collagen^(+/+)^ in 3D (**h**). Incomplete decellularization was observed for group IV well as occurrence of few cells^(+/−)^ (**d**) and fragmented matrix (3D) (**i**). Group V has presence of cells^(+/−)^ in scattered form within tissue matrix^(+/−)^ (**e**, **j**) seen in 3D

**Fig. 3:**
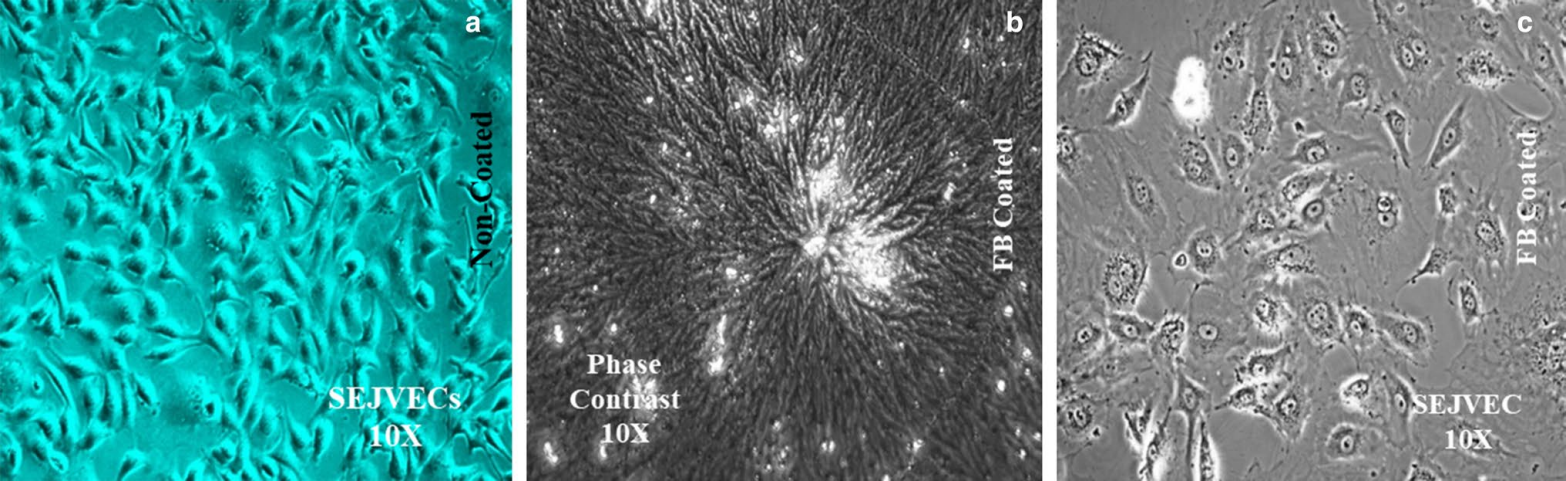
Endothelial cells phenotype on non-coated FG^(−/−)^, FG^(+/+)^ coated surfaces and 3D imaging. Phenotypic cobble stone morphology was observed for SEJVECs (**a**) on non-coated FG^(−/−)^ surface. However, SEJVECs on FG^(+/+)^ coated surfaces showed spreading cellular lamellas and filopodia along with cell to cell adherence (**c**) with clear resemblance of crater-like cellular nucleus. The coating surface of FG^(+/+)^ showed meshwork organization in a whirlpool orientation (**b**)

**Fig. 4:**
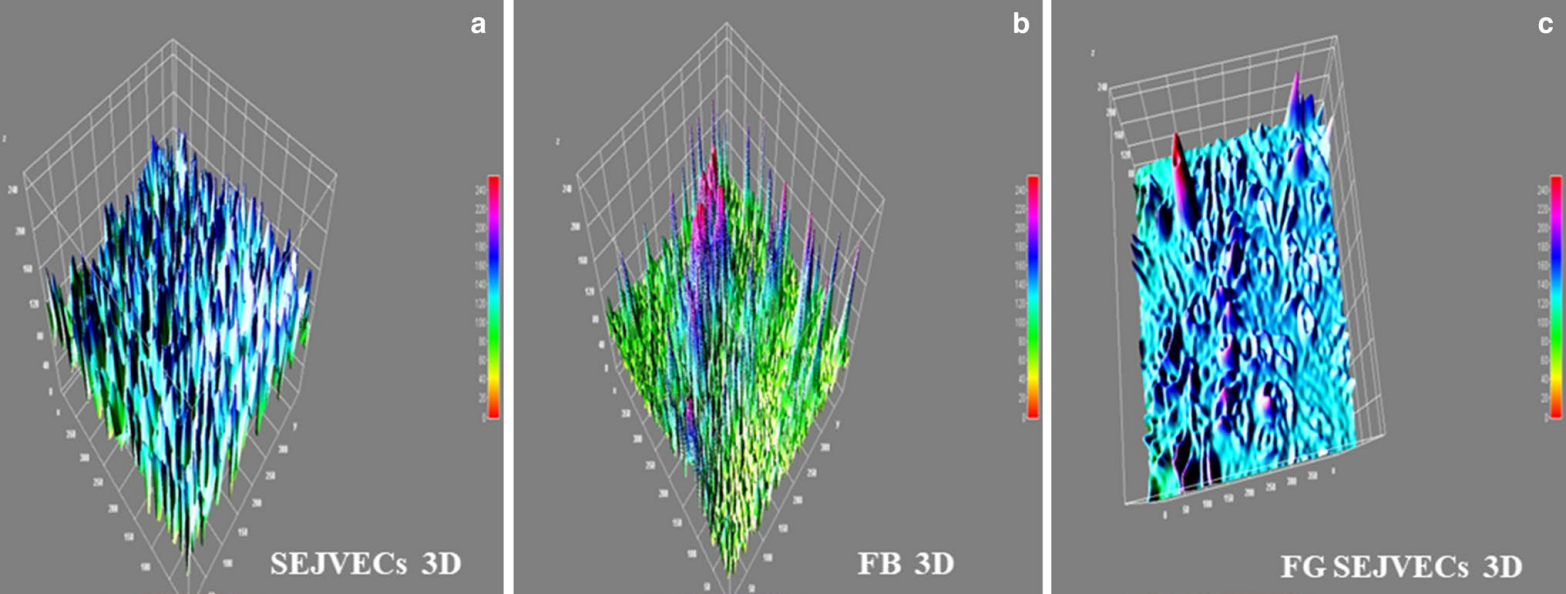
Image J 3D construction gave the mechanism about the plasticity of SEJVECs on FG^(−/−)^ and FG^(+/+)^ coated surfaces (**a**, **c**). 3D image construction also provided us the inter-nodal arrangement (meshwork) of fibrin fibrils (**b**)

**Fig. 5:**
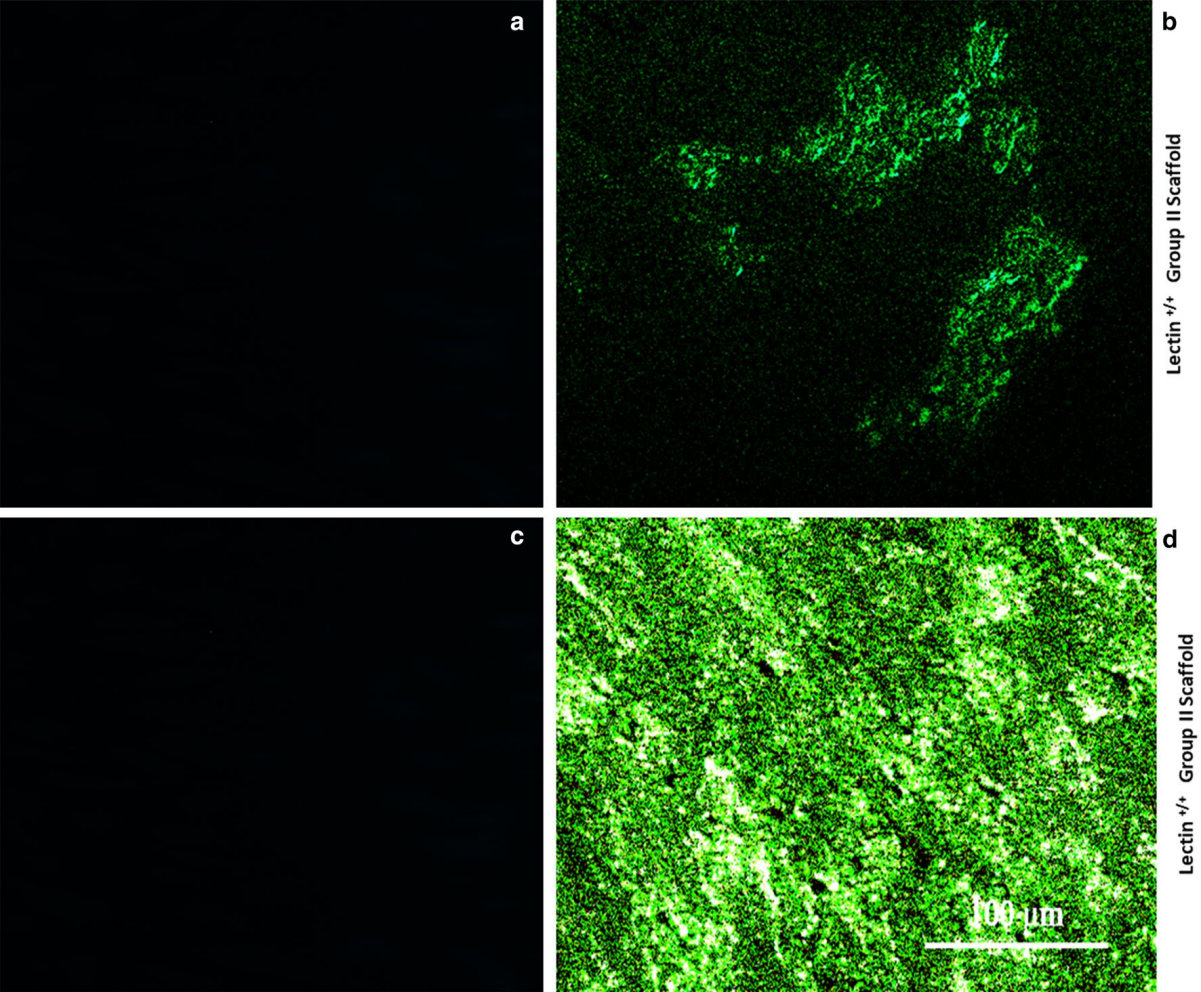
Molecular marker expression. The SEJVECs expanded onto the scaffolds does not change the lectin^(+/+/+)^ phenotype expression (**b**). Further expansion of SEJVECs on the group II scaffolds does not alter the endothelial specific universal molecular marker vWF^(+/+/+)^ expression and this clearly demarcates the basal lamina coated with FG^(+/+)^ does not trans-differentiate the endothelial cells to any other lineage (**d**)

**Fig. 6:**
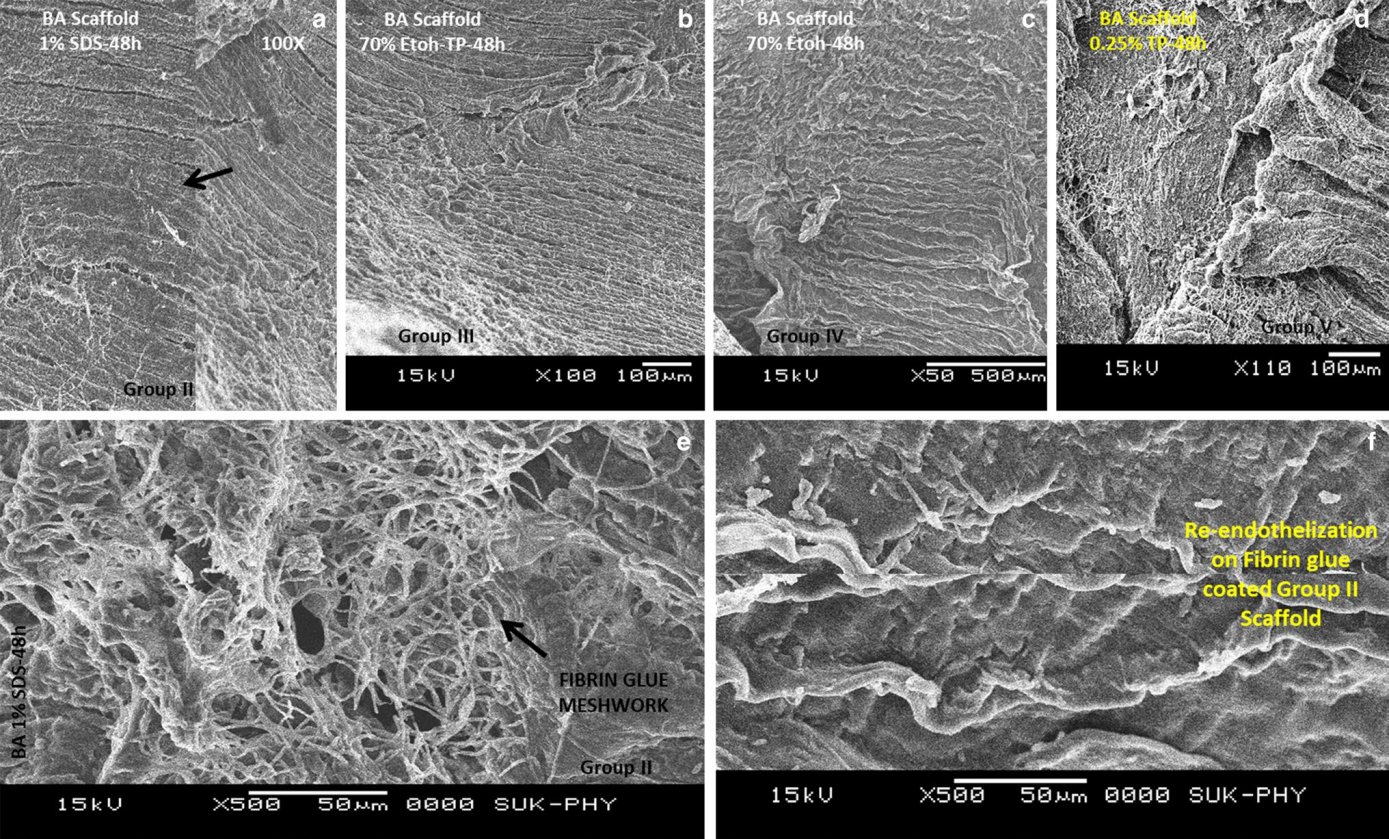
Scanning electron microscopy (SEM) and Image J 3D construction. Scanning electron microscopy (SEM) for group II (**a**), III (**b**), IV (**c**) and V (**d**) scaffolds showed the presence of basal lamina with absence of endothelium. SEM imaging for group II FG^(+/+)^ coated surface showed meshwork inter-nodal spaces (**e**). Further, re-endothelialization (SEJVECs) on FG^(+/+)^ coated basal lamina shows remolding and plasticity of the cells (**f**)

**Fig. 7:**
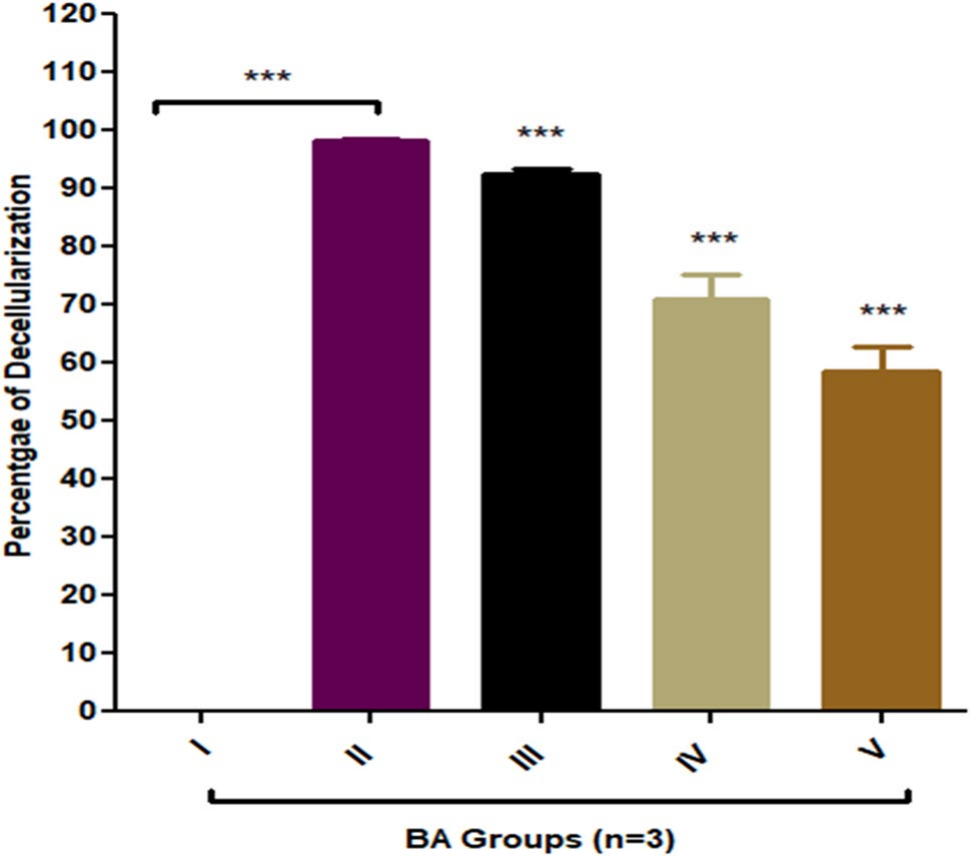
Percentage of decellularization for BA groups II, III, IV and V. Maximum percentage of decellularization was observed for group II (1% SDS) and showed one-way analysis of variance (ANOVA) ***p < 0.05 level of significance. Group III, IV and V also showed ***p < 0.05 significant level however, decellularization percentage was less in comparison to group B. All decellularised groups were compared with non-decellularised group I (Dunnett’s test)

### Tensile testing BA scaffolds

**Fig. 8:**
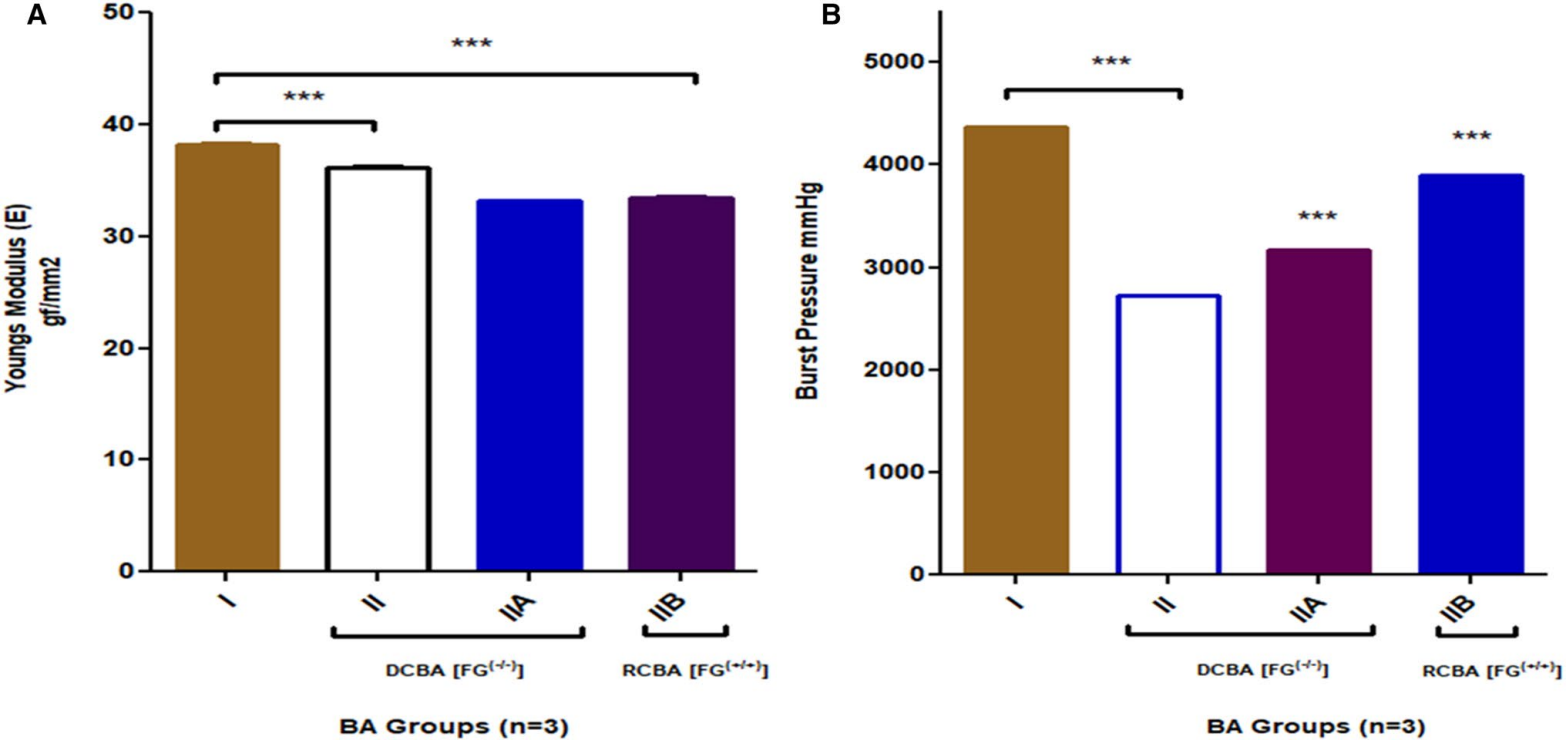
**a** Young’s modulus/elasticity showed significant decrease for groups II A and B (***p < 0.05) in comparison to I group. There was significance (ns) for one-way analysis (ANOVA) between group I and IIA. However, re-cellularized (RCBA) group IIB showed recover of elastic properties and has a significant one-way analysis (ANOVA) (***p < 0.05) in comparison to group I. All the decellularized and re-cellularized (RCBA) were compared with group I for elastic (Young’s modulus) properties (Dunnett’s test). **b** Burst pressure (mmHg) measured showed significance for one-way analysis of variance (ANOVA) ***p < 0.05 for group II B in direct comparison to group I. The decellularised groups also showed significance level in comparison to group I. However, the recellularized (RCBA) groups IIA and B also showed one-way analysis (ANOVA) ***p < 0.05 significant levels in comparison to group I

The mechanical tensile testing for maximum displacement strain (mm) showed the one-way analysis of variance (ANOVA) ***p < 0.05 for groups I, II, IIA, IIB there was significance of variance between group I and II including IIA and B. Young’s modulus (elasticity) and burst pressure showed the one way of variance (ANOVA) ***p < 0.05 for all groups (Fig. 8a, b). Group II B scaffold without cell and SEJVECs scaffold therefore appeared to hold promises for in vivo performance closely resembling physiological norms. After biocompatibility testing, retention of biomechanical tensile properties was confirmed (Fig. 8a, b). So the biomechanical tensile properties for re-endothelialized group IIA also showed conformational regain.

### DNA quantification assay

**Fig. 9:**
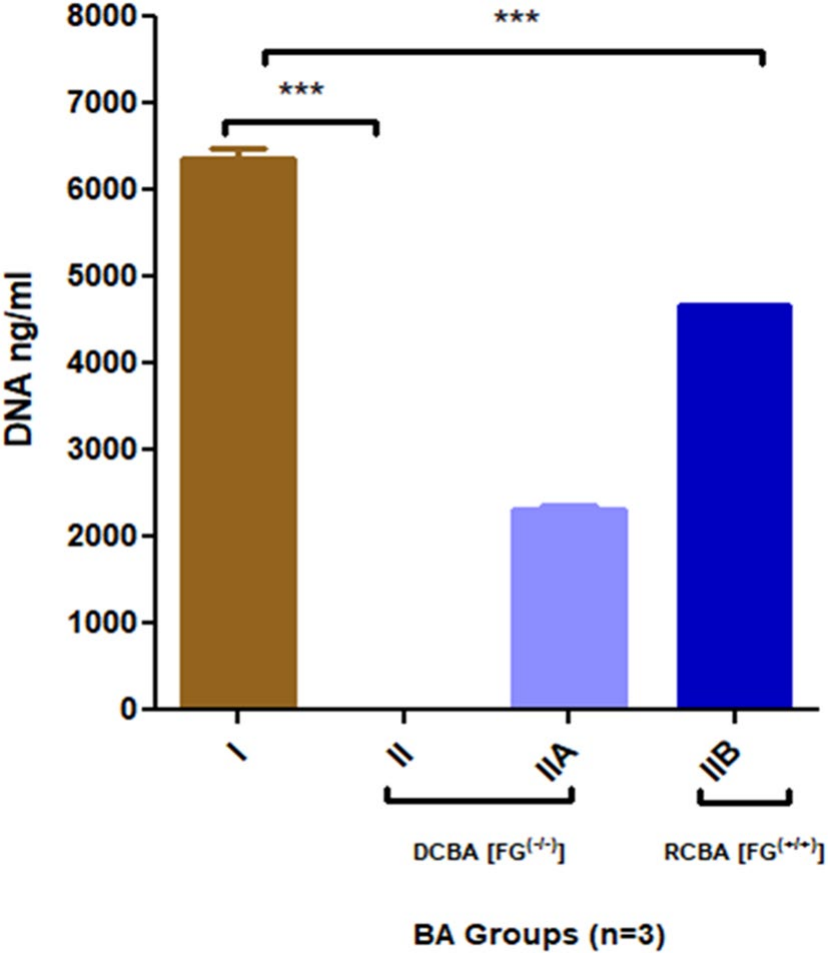
PicoGreen DNA ng/mL analysis showed complete absence of host cellular genetic material for group II and showed one-way analysis of variance (ANOVA) ***p < 0.05 level of significance. However, recellularized (RCBA) (group IIB) also showed increase in DNA ng/mL in comparison to decellularised scaffold (***p < 0.05)

The PicoGreen analysis indicated the presence or absence of residual host DNA in the decellularized BA tissue, helping to select group scaffolds and re-endothelization (SEJVECs) of the intima lumenal surface. Our results indicated that group II (1% SDS) revealed complete absence of DNA in comparison to group I (6368 ± 200.6). Hence, only group II decellularized bovine aorta (DCBA) scaffold was selected for further endothelial cell expansion studies (Graph 3) [0 ng/mL ± 0]. Further, cellular migration showed the presence of DNA on recellularized bovine aorta (RCBA) group IIA (2319.67 ± 53.29) and group IIB (4680.67 ng/mL ± 0.5) confirming the presence of cellular genetic material (Fig. 9).

### MTT assay for HSVECs expansion on scaffold

**Fig. 10:**
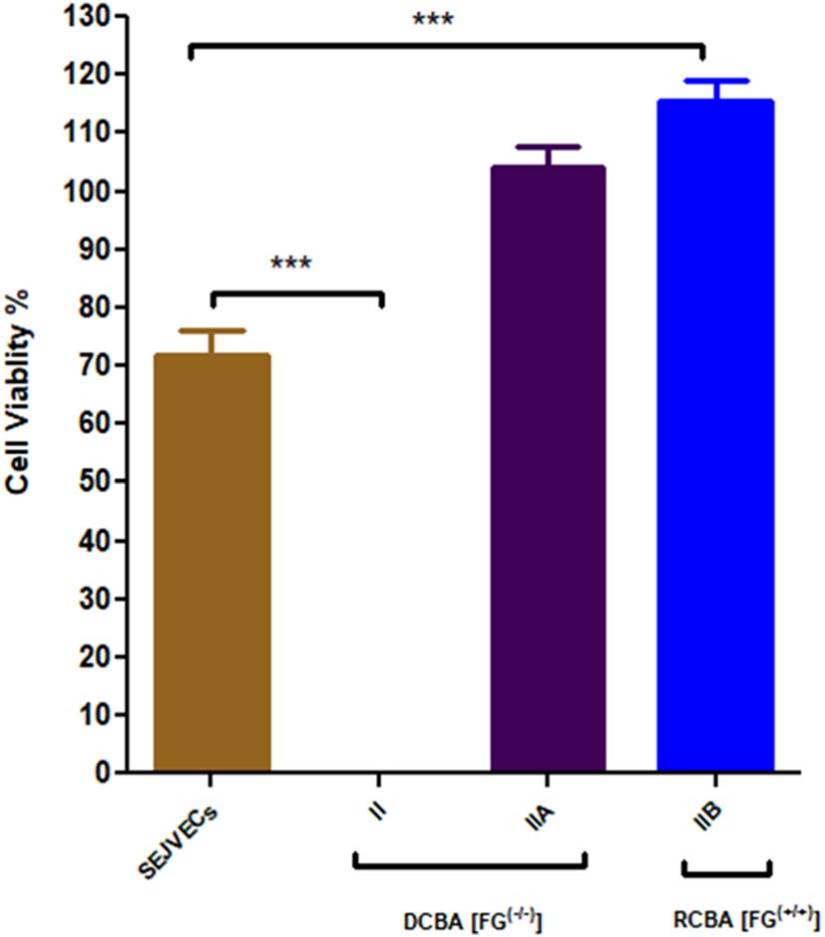
Cell viability assay showed expansion of SEJVECs on group II A and IIB scaffold. One-way analysis of variance (ANOVA) ***p < 0.05 showed level of significance between SEJEVCs in culture and SEJVECs expanded on group IIA and group IIB scaffolds (Dunnett’s test)

SEJVECs proliferation for the group IIA (1% SDS) scaffold was confirmed by MTT cell viability assay (Fig. 10). The formazan crystals were assessed in the proliferation SEJVECs in culture (control, *n* = 5) as well as onto the group II A scaffold. The MTT assay showed that SEJVECs proliferation was greater for group II A and B (1% SDS) (*n* = 5) than the culture. SEJVECs proliferation on 1 cm in group II was about 500% and HSVECs in the cell culture was about 90%. This showed that HSVECs proliferation rate [(mean % ± SD) 115% ± 6] is greater for group IIB in comparison to tissue culture plate surface control [(mean % ± SD) 71% ± 7.6].

## Discussion

The novelty of our study is to use a combination of methods to fabricate a metabolically active, non-thrombotic, re-endothelized, large diameter scaffold from xenogeneic source. Researchers made use of polymers for fabrication of grafts depending upon the porosity and patency. The acceptability of biopolymers varied and it was found that there was a problem related to gaseous exchange, cellular infiltration and migration involving graft adsorption into host tissue (Campbell et al. 1975). The clinicians using the biografts of large diameter were not to the mark of compliance resulting in failure and rejection due to central necrosis (Campbell et al. 1975). Cross-linked bioimplants from xenogeneic origin without toxicity neutralization were used previously as a conduit. The implants faced the problem of foreign body reaction, failure to endothelize, thrombus formation and calcification (Ibanez et al. 2007). Few researchers used a decellularization technique followed by re-endothelization without using any biological sealants. However, the complete re-endothelization was not achieved resulting in denudation zones and the thrombogenesis (Campbell et al. 1975). Sealants used for re-endothelization were gelatin, fibronectin, collagen but the adherence of the endothelial cells as per the surface alignment was not achieved as well as the cells could not withstand the shear stress (dyne/cm^2^) (Harrison 1958).

In our study, we used a large diameter xenogeneic blood vessel (aorta) instead of cross-linked conduit, which was decellularized using different chemicals. We compared the percentage of decellularization for all the groups (Table 1). It was found that maximum percentage of decellularization was achieved in group II. The histological findings showed that complete absence of cells without alteration of extracellular matrix was more promising with group II scaffolds (Table 2). The results for other groups showed lesser percentage of decellularization along with more or less alteration in collagen and matrix (Table 2). The contemporary 2D images observed in histological examination have limitations in understanding the cellular organization and their plasticity with respect to extracellular matrix. To overcome this hurdle, we used image J software for 3D constructions of histological pictures. All the group images were compared with group I images. Image J construction also resolved the 2D cell cultural difference in FG^(+/+)^ and FG^(−/−)^ (Fig. 4a, c). Phase contrast imaging showed the phenotypic plasticity as well as remolding of cells on FG^(+/+)^ scaffolds which was also proven by image J constructs. SEM imaging of the FG^(+/+)^ scaffolds showed internodal distance and meshwork. However, image J constructs of the same meshwork showed various track-like alignments supporting endothelial cells migration. Biomechanical tensile testing showed the decrease in elasticity in group II. Although the regain of elasticity was not observed for FG^(+/+)^ and FG^(−/−)^ re-endothelized grafts (Fig. 8a). However, the burst pressure was increased for both groups (Fig. 8b).

There was an absence of host tissue specific DNA which was analyzed by PicoGreen DNA assay. The re-endothelized FG^(−/−)^ and FG^(+/+)^ scaffolds shows increase in DNA content (Table 4). The Graph 3 clearly demarcate that the FG^(+/+)^ scaffolds supports significant cellular DNA synthesis in comparison with FG^(−/−)^ scaffolds. Figure 10 emphasizes that the FG^(−/−)^ and FG^(+/+)^ scaffolds for group II are non-toxic and supports the rapid endothelization and proliferation. Further, cell viability assay showed that the FG^(+/+)^ scaffolds hold higher proliferation in comparison to FG^(−/−)^ scaffolds (Fig. 10). FG^(+/+)^ re-endothelized scaffold signifies biocompatibility, biomimetic properties of large diameter blood vessel (Fig. 10).

**Table 4:** Cell viability, DNA quantification, elasticity

Sample	Cell viability (%)	DNA (ng/mL)	Elasticity (Young’s modulus) (gf/mm^2^)	Burst pressure (mmHg)	*p* value
Group I	71.0	6368.33	38.18	4371	***p < 0.05
Group II A FG^(−/−)^	104.2	2319.67	33.13	3175	
Group II B FG^(+/+)^	115.66	4680.67	33.4	3891	

The limitation of this study was that the re-endothelized graft implantation was not performed in higher animal models. The animals’ studies related to fabrication of larger diameter aorta and their implants could give us an insight about the hemodynamics, rheology towards the graft patency and compliances. In future, this biocompatible, biomimetic re-endothelized scaffold can be planned for non-clinical followed by clinical trials.

## Conclusion

Our study concludes that larger diameter scaffolds can be fabricated using chemicals such as SDS and using fibrin glue sealant supports enhanced re-endothelization. This study establishes the various comparative approaches in fabrication of large diameter blood vessel so as to select a closer biomimetic and biocompatible scaffold to the native origin which can be a better option for the futuristic vascular surgeries.

## Abbreviation

BA: Bovine aorta
SEJVECs: Sheep external jugular vein endothelial cells
ECM: Extracellular matrix
vWF^(+/+)^: Von Willebrand factor expression
Lectin^(+/+)^: Lectin expression
SEM: Scanning electron microscopy
SDS: Sodium dodecyl sulphate
EVG: Verhoeff–van Gieson
HE: Hematoxylin–eosin
FG^(−/−)^: Fibrin glue non-coated
FG^(+/+)^: Fibrin glue coated
Elastin and collagen: Absent (−/−); present/absent (+/−); moderately present (+/+); extensively present (+/+/+)
